# Regression Modeling of the Antioxidant-to-Nephroprotective Relation Shows the Pivotal Role of Oxidative Stress in Cisplatin Nephrotoxicity

**DOI:** 10.3390/antiox10091355

**Published:** 2021-08-26

**Authors:** Alfredo G. Casanova, Mykola Harvat, Laura Vicente-Vicente, Óscar J. Pellicer-Valero, Ana I. Morales, Francisco J. López-Hernández, José D. Martín-Guerrero

**Affiliations:** 1 Institute of Biomedical Research of Salamanca (IBSAL), 37007 Salamanca, Spain; alfredogcp@usal.es (A.G.C.); lauravicente@usal.es (L.V.-V.); amorales@usal.es (A.I.M.); flopezher@usal.es (F.J.L.-H.); 2 Department of Physiology and Pharmacology, University of Salamanca, 37007 Salamanca, Spain; 3 Toxicology Area, University of Salamanca, 37007 Salamanca, Spain; 4 Fundación Instituto de Estudios de Ciencias de la Salud de Castilla y León, 42002 Soria, Spain; 5 Group of Translational Research on Renal and Cardiovascular Diseases (TRECARD), National Network for Kidney Research REDINREN, RD016/0009/0025, Instituto de Salud Carlos III, 37007 Salamanca, Spain; 6 Intelligent Data Analysis Laboratory (IDAL), Dpt. Enginyeria Electrònica, ETSE-UV, Universitat de València, 46100 Valencia, Spain; Mykola.Harvat@uv.es (M.H.); oscar.pellicer@uv.es (Ó.J.P.-V.); 7 Group of Biomedical Research on Critical Care (BioCritic), Valladolid University Hospital, 47003 Valladolid, Spain; 8 Disease and Theranostic Modelling (DisMOD) Working Group, IBSAL, 37007 Salamanca, Spain

**Keywords:** cisplatin, nephrotoxicity, prevention, antioxidants, preclinical, linear fit

## Abstract

The clinical utility of the chemotherapeutic drug cisplatin is significantly limited by its nephrotoxicity, which is characterized by electrolytic disorders, glomerular filtration rate decline, and azotemia. These alterations are consequences of a primary tubulopathy causing injury to proximal and distal epithelial cells, and thus tubular dysfunction. Oxidative stress plays a role in cisplatin nephrotoxicity and cytotoxicity, but its relative contribution to overall toxicity remains unknown. We studied the relation between the degree of oxidative reduction (provided by antioxidant treatment) and the extent of nephrotoxicity amelioration (i.e., nephroprotection) by means of a regression analysis of studies in animal models. Our results indicate that a linear relation exists between these two parameters, and that this relation very nearly crosses the value of maximal nephroprotection at maximal antioxidant effect, suggesting that oxidative stress seems to be a pivotal and mandatory mechanism of cisplatin nephrotoxicity, and, hence, an interesting, rationale-based target for clinical use. Our model also serves to identify antioxidants with enhanced effectiveness by comparing their actual nephroprotective power with that predicted by their antioxidant effect. Among those, this study identified nanoceria, erythropoietin, and maltol as highly effective candidates affording more nephroprotection than expected from their antioxidant effect for prospective clinical development.

## 1. Introduction

**Figure 1:**
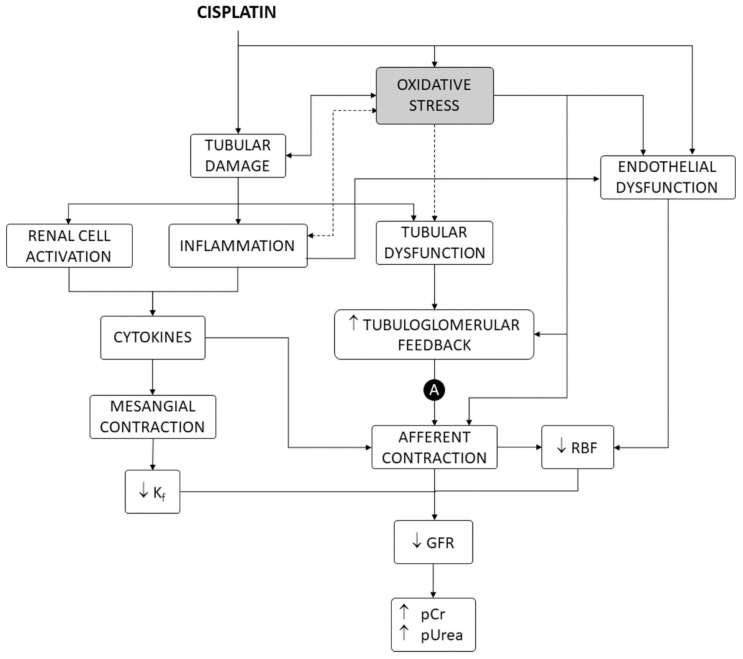
Mechanisms of cisplatin nephrotoxicity, including oxidative stress as a contributing factor. A, autoregulation of renal blood flow and intraglomerular blood pressure. GFR, glomerular filtration rate. Kf, ultrafiltration coefficient. pCr, plasma creatinine concentration. pUrea, plasma urea concentration. RBF, renal blood flow.

Cisplatin is one of the most potent and widely used chemotherapeutic drugs for the treatment of a variety of solid cancers [1], but its dosage and clinical utility are limited by nephrotoxicity [2]. Nephrotoxicity occurs in 25–35% of adult [3] and 70% of pediatric [4] therapeutic courses. Direct effects on the renal vasculature are involved [2], but cisplatin nephrotoxicity mostly shows a tubular damage pattern of dysfunction and derangement, producing electrolytic disturbances (i.e., most typically hypomagnesemia and hypokalemia), acute tubular injury (ATI), and acute kidney injury (AKI), with elevated plasma creatinine (pCr) and urea (pUrea) levels [2,5,6,7], which may occasionally progress to chronic fibrotic nephropathy [8,9]. As shown in Figure 1, tubular damage causes a reduction in glomerular filtration rate (GFR) by a number of mechanisms, including activation of the tubuloglomerular feedback (TGF) mechanism and renal vasoconstriction induced by inflammation and factors released by activated renal cells [2,10].

This pathophysiological pattern results from cisplatin accumulation in proximal (mainly the S3 segment) [11,12] and distal tubule cells [2,13], which causes diverse cellular alterations, chiefly including inhibition of membrane transporters [2,14], interference with metabolic pathways [15], and cell death [16,17]. Tubular cell death shows apoptotic and nonapoptotic phenotypes, depending on the level of exposure to cisplatin [16]. While lower concentrations induce apoptosis, higher concentrations cause a necrotic-like phenotype [18,19]. Inside the cells, cisplatin becomes aquated and turns into a potent nucleophilic that binds to numerous targets, most prominently nucleic acids and many proteins [18,20]. Cisplatin cytotoxicity has been traditionally explained by formation of inter- and intra-strand adducts with nuclear DNA, which activates DNA repair mechanisms that, when overwhelmed, in turn, activate apoptosis. This cytotoxic mechanism is very effective in rapidly dividing cells, because nonrepaired DNA activates the p53–p21 cyclin-dependent kinase 2 (cdk2) pathway to make death/life decisions at cell division checkpoints [21].

Despite bearing a high and ready division capacity (for regeneration purposes), the proliferation rate of tubular epithelial cells is, however, very low under normal conditions [22]. Cell-cycle-independent mechanisms have been described, which might explain cisplatin cytotoxicity in target, nonproliferating epithelial cells, in which the drug accumulates [2,13]. Apoptotic and necrotic signaling is induced from damaged structures and organelles, such as mitochondria, endoplasmic reticulum, lysosomes, and others [16,21]. Cisplatin also induces oxidative stress in tubule epithelial cells in culture and in animal models [23,24,25] by accumulating in mitochondria and interfering with mitochondrial homeostasis and respiration [16,23]. Oxidative stress causes, or contributes to causing cell death, in general [26,27], and specifically after exposure to cisplatin [28,29,30]. In addition, oxidative stress also participates in other mechanisms of nephrotoxicity, such as renal vascular [31,32,33,34] and mesangial [35] contraction, endothelial dysfunction [36,37], inflammation [38,39,40], and TGF enhancement [32,33], leading to renal blood flow and GFR reduction and damage amplification [2,21] (Figure 1).

Oxidative stress has been proposed as a prominent event and mediator of cisplatin cytotoxicity and nephrotoxicity [2,13,21], but its relative weight among other pathophysiological mechanisms, and its hierarchical and causality relation with them, are mostly unknown. In this article, we studied and modeled the relation between the degree of reduction in oxidative stress and the degree of protection of cisplatin nephrotoxicity bestowed by exogenous antioxidants in a number of studies with animal models. A key role of oxidative stress in cisplatin nephrotoxicity was inferred from the linear relation between the antioxidant and nephroprotective effects, with almost complete prevention of nephrotoxicity at maximal antioxidant effect.

## 2. Materials and Methods

### 2.1. Data Mining

The data used for this study were obtained from the literature search carried out in our previous meta-analysis [41], in which preclinical studies reporting molecules or products preventing cisplatin nephrotoxicity were identified. Among them, only those articles meeting the following criteria were used: (1) evaluating antioxidant nephroprotectants, (2) conducted on experimental animals, (3) providing number of individuals per experimental group, (4) using cisplatin as the nephrotoxic agent, (5) written in English, (6) fully accessible for authors (through journal subscriptions, request to authors, or open access), (7) using pUrea or blood urea nitrogen (BUN) level as the parameter to estimate nephroprotection, and (8) using malonyldialdehyde (MDA) to evaluate oxidative stress, as previously described [42]. The subsequent mathematical analysis was performed only with those studies reporting statistically significant nephroprotective and antioxidant effects (with respect to the group that received cisplatin and no nephroprotectant). Publication bias was evaluated with the asymmetry tests of Begg and Mazumdar [43], and Egger et al. [44]. 

### 2.2. Mathematical Modeling

With the objective of evaluating a potential relation between the antioxidant and the nephroprotective activity of the nephroprotectants included in the study, the following parameters were defined:

- Nephroprotection index (*E_nep_*):

$$E_{nep}=1-\frac{MaxNP-BasNP}{MaxNA-BasNA}$$

where *MaxNP* is the value of the nephrotoxicity biomarker (i.e., pUrea or pBUN) at the maximum toxicity time in the nephroprotectant+cisplatin group; *BasNP* is the value of the nephrotoxicity biomarker at basal time point in the nephroprotectant+cisplatin group; *MaxNA* is the value of the nephrotoxicity biomarker at the maximum toxicity time point in the cisplatin group; and *BasNA* is the value of the nephrotoxicity biomarker at the basal time point in the cisplatin group. Thereof, *MaxNP-BasNP* corresponds to the increment in the level of the nephrotoxicity biomarker in the nephroprotectant+cisplatin group; and *MaxNA-BasNA* corresponds to the increment in the level of the same nephrotoxicity biomarker in the aminoglycoside group. *E_nep_* > 0 denotes nephroprotection (i.e., reduced cisplatin nephrotoxicity due to the action of the nephroprotectant), with the higher the value of *E_nep_*, the higher the nephroprotective effect. *E_nep_* = 1 represents total nephroprotection. *E_nep_* = 0 means there is no effect exerted by the nephroprotectant.

- Antioxidant index (*E_oxi_*):

$$E_{oxi}=1-\frac{MaxOP-BasOP}{MaxOA-BasOA}$$

where *MaxOP* is the value of the oxidative stress biomarker (i.e., MDA) at the maximum toxicity time point in the nephroprotectant+cisplatin group; *BasOP* is the value of the oxidative stress biomarker at the basal time point in the nephroprotectant+cisplatin group; *MaxOA* is the value of the oxidative stress biomarker at the maximum toxicity time point in the cisplatin group; and *BasOA* is the value of the oxidative stress biomarker at the basal time point in the cisplatin group. Thereof, *MaxOP-BasOP* corresponds to the increment in the level of the oxidative stress biomarker in the nephroprotectant+cisplatin-treated group; and *MaxOA-BasOA* corresponds to the increment in the level of the same oxidative stress biomarker in the cisplatin-treated group. *E_oxi_* > 0 denotes antioxidant activity due to the protector, with the higher the value of *E_oxi_*, the higher the antioxidant effect. *E_oxi_* = 1 represents a complete antioxidant effect. *E_oxi_* = 0 means no antioxidant effect is exerted by the nephroprotectant.

The *E_oxi_* versus *E_nep_* relation was represented. We used an ordinary least squares (OLS) approach for building a linear regression model between *E_oxi_* as an independent variable, and *E_nep_* as a dependent variable. Nonlinear models were also taken into account, but they did not improve the performance achieved by their linear counterparts. As on the basal state of *E_oxi_* = 0, we should expect no nephroprotection effect (*E_nep_* = 0); we used this fact in the model assessment and supposed a zero-centered model that was tested with a proper model assessment using the Akaike information criterion (AIC). In particular, the final linear regression model was given by a weighted linear combination:

$$E_{nep}=w*E_{oxi}$$

The model was assessed by measuring the statistical significance of the coefficients; the variability of the relationship between the predictors and the target value was determined by the corresponding $R^{2}$ coefficient [45].

As the presence of outliers was considerably high, two additional robust techniques were considered, namely the Huber regression and the random sample consensus (RANSAC) algorithm [46,47]. The Huber regression is a robust technique that uses a Huber loss function instead of the standard least squares in order to penalize the error depending on their magnitude [47]. RANSAC is an iterative estimation algorithm which fits several iterative models on subsets of data, and then selects the subset with the least average error that, by assumption, is the subset with no outlier points [46]. The value of the slope coefficient corresponding to each of the three models was eventually compared as an evaluation metric about the influence of outliers in the coefficient estimation carried out by OLS. All three models were fitted using Python module Scikit-learn [48]; the rest of the processing was performed in Python programming language [49].

## 3. Results

The characteristics of the studies included in this work are provided in Table 1.

The Begg–Mazumdar test applied to assess potential publication bias yielded a Kendall’s tau of 0.74 (*p* < 0.001). Similarly, the Egger test provided a bias of 10.49 (95% CI = 9.56, 11.43; *p* < 0.001). Both tests showed the presence of asymmetry. However, in our study, this result was expected and is not necessarily reflective of publication bias. In fact, pursuant of our objective, only studies reporting a statistically significant nephroprotective effect were included, as stated in the Methods.

The OLS model was evaluated by checking the statistical significance of the coefficients with an alpha error threshold of 0.01. We obtained the following results: *w =* 0.938 (95% CI = (0.89, 0.987), *p* < 0.0001). We used the Akaike information criterion (AIC) [112] to assess the choice of including or excluding the bias term from the final model. We obtained an AIC_intercept_ = −66.32 for the linear model with bias term, and AIC_base_ = −61.90 for the model without bias term. Therefore, based on this result, we only kept the slope term in the resulting model, *R^2^* = 0.932, meaning that 93.2% of the variability of $E_{nep}$ could be explained by $E_{oxi}$.

**Figure 2:**
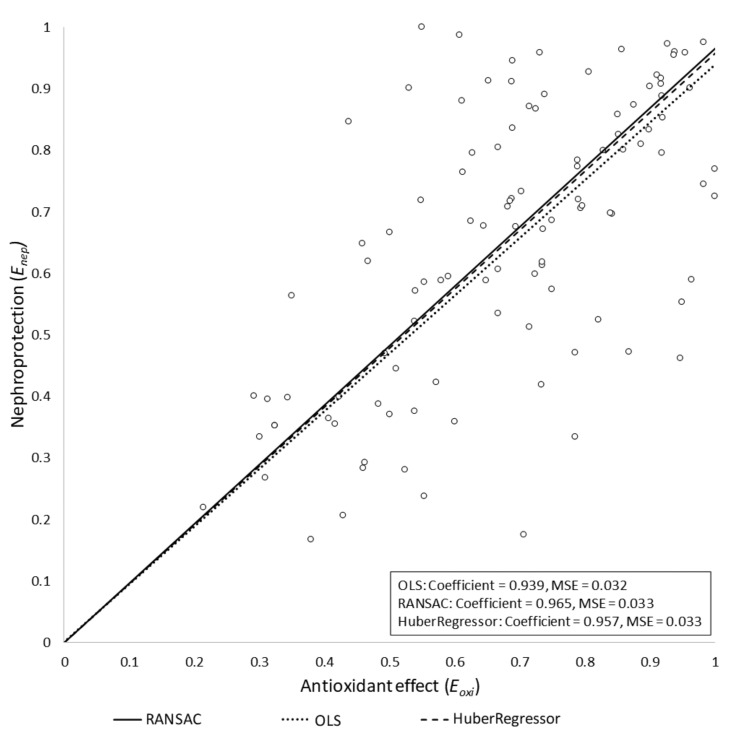
Regression results for the three tested models. The nephroprotection index (*E_nep_*) is represented versus the antioxidant index (*E_oxi_*) for the OLS, Huber regression, and RANSAC regression models, all of them yielding similar results. The RANSAC model provided the most robust fit, as it empirically ignored some outlier points, giving them a zero weight in the final adjustment. MSE, mean squared error.

The potential influence of outliers in the final model was also assessed. In particular, Huber and RANSAC regressions were obtained, assuming the same model as for the OLS case (i.e., without intercept term). Both algorithms yielded similar slope values to that obtained by the OLS regression model: *w_Huber_ =* 0.957 and *w_RANSAC_* = 0.965. We concluded that, under our assumptions, the outliers had no significant influence on the final model. The three models are depicted in Figure 2. Studies in which *E_oxi_* > 1 were removed from the models. In these studies, the antioxidant reduced oxidative stress beyond the basal level (i.e., the level of oxidative stress in the control group), which had a negative impact on nephroprotection. Specifically, *E_nep_* showed a negative slope beyond *E_oxi_* = 1 (data not shown). This is because normal (i.e., basal) production of reactive oxygen species (ROS) has been shown to have homeostatic signaling roles [113,114,115]. As a corollary, inhibition of basal ROS production may reasonably result in deleterious effects for cell and organ function [116].

**Figure 3:**
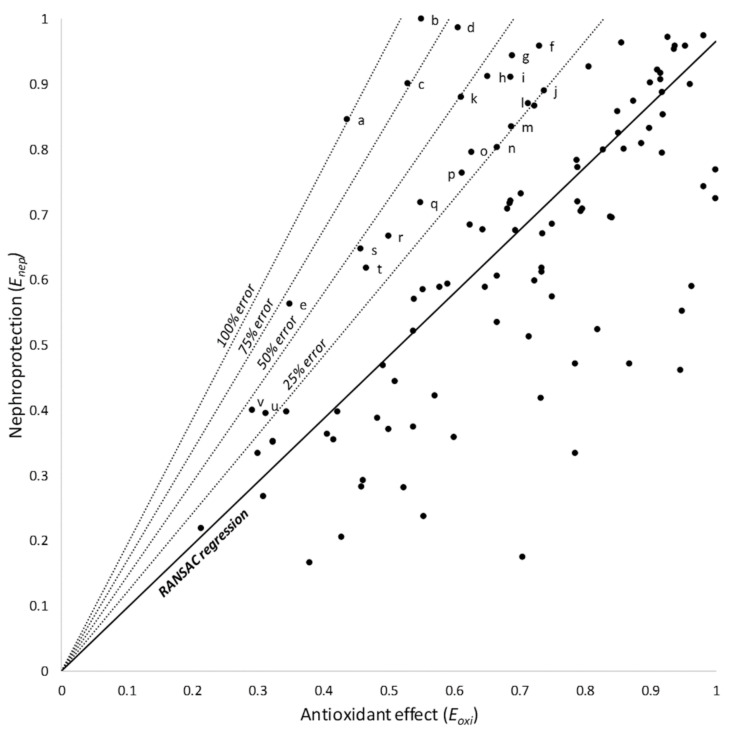
RANSAC linear regression and 25, 50, 75, and 100% relative error areas. Products over the model afford a greater nephroprotection than expected from their antioxidant effect. Products within these areas are identified and shown in Table 2. Letters (a through v) identify individual products, as listed in Table 2.

Products located over the model provided more nephroprotection than expected from their antioxidant effect. Based on the RANSAC regression (the model with the most robust fit), these products were subclassified as showing 25, 50, 75, or 100% of additional nephroprotection (Figure 3); they are identified and listed in Table 2.

## 4. Discussion

The regression model best adjusting our experimental data shows a linear relationship between inhibition of oxidative stress and amelioration of cisplatin nephrotoxicity (Figure 2). This relation intercepts the nephroprotection axis (i.e., the *y*-axis) very near the *E_nep_* = 1 value at the maximal antioxidant point (i.e., *E_oxi_* = 1 in the *x*-axis). This indicates that a complete abrogation of oxidative stress apparently leads to a complete prevention of nephrotoxicity. Thus, oxidative stress might not only be a contributing, but a pivotal mechanism of cisplatin nephrotoxicity. Cisplatin nephrotoxicity is a tubulopathy, in which all pathophysiological and clinical manifestations derive from cytotoxic tubular injury as the primary event (Figure 1) [2,13]. Consequently, oxidative stress must also be in the core of cisplatin cytotoxicity. 

Mitochondria are the main intracellular site of cell life/death decision [117,118,119]. Mitochondria funnel and integrate stress signals arising from damaged subcellular structures and organelles, including themselves, and activate apoptotic and necrotic death programs that mostly pose no-return points for cell demise [120]. One of these signals is oxidative stress. Extramitochondrial sources of ROS exist (e.g., the cytosol and the endoplasmic reticulum) [121], but mitochondria are the main source of ROS production and overproduction [115]. Mitochondrial outer membrane permeabilization (MOMP) is a mandatory event for the release of proapoptotic factors (e.g., cytochrome c and AIF), apoptosome formation in the cytosol, and initiation of intrinsic apoptosis [119]. Intracellular death signals regulate MOMP by targeting the outer membrane through pro- and anti-apoptotic Bcl-2 family members, which directly modulate its permeability [118,122]. Inner membrane permeabilization (i.e., mitochondrial permeability transition, MPT) is also intimately related to cell death. MPT is bidirectionally linked to transmembrane mitochondrial potential (∆Ψ) dissipation, and causes intermembrane swelling, outer membrane disruption, and MOMP. MPT is mediated by a multiprotein complex, the permeability transition pore (PT pore or PTP). PTP is located at sites of inner–outer membrane connections (where Bcl-2 family members accumulate), is inhibited by anti-apoptotic Bcl-2 members, is critical for apoptosis, and participates in MOMP [123,124,125].

**Figure 4:**
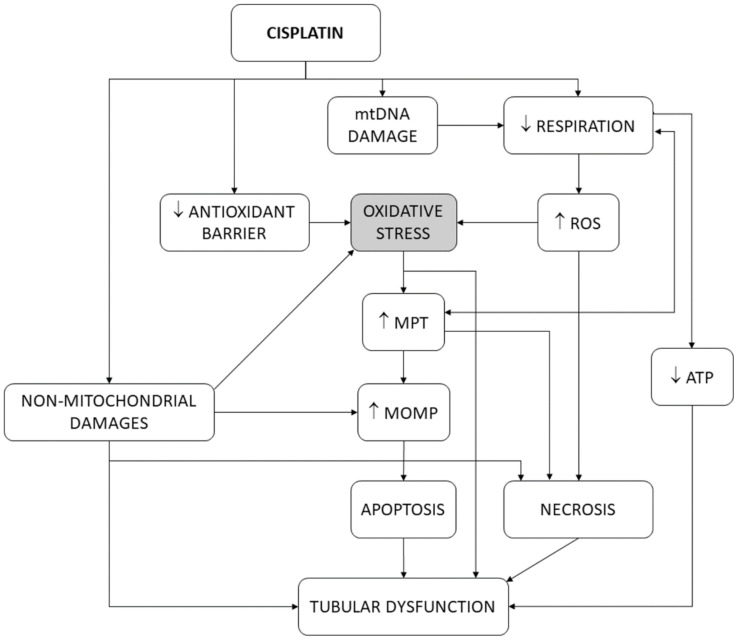
Schematic depiction of the pivotal role of oxidative stress in the tubular dysfunction induced by cisplatin. ATP, adenosine triphosphate. MOMP, mitochondrial outer membrane permeability. MPT, mitochondrial permeability transition. mtDNA, mitochondrial DNA. ROS, reactive oxygen species.

In isolated mitochondria [126], cisplatin interferes with the respiratory chain, produces oxidative stress [127] and rapid cytochrome c release [128], and causes calcium-dependent mitochondrial swelling and mitochondrial depolarization, as a consequence of PT pore opening [129]. In this scenario, oxidative stress may be the cause or the consequence of the other events. In fact, decoupling or inhibition of mitochondrial respiration induces both PT pore opening and oxidative stress [117,130,131]. PT pore opening (and, thus, MPT) is triggered by mitochondrial Ca^2+^ and potentiated by oxidative stress [125,132,133], suggesting that alterations in respiration induce oxidative stress, and this, in turn, contributes to the opening of the PT pore. In agreement, antioxidants inhibit MPT [134]. However, vice versa is also possible: PT pore opening produces ∆Ψ dissipation, respiratory uncoupling, and oxidative stress [124,132,135]. As such, oxidative stress and mitochondrial dysfunction induce one another [136,137], and so a causality dilemma existed for cisplatin cytotoxicity [16]. Cisplatin also causes oxidative stress by directly damaging mitochondrial DNA (mtDNA) [128,138,139,140], which impairs appropriate expression of mitochondrial enzymes forming the respiratory chain, and thus induces oxidative stress. Finally, cisplatin abates the antioxidant barrier by inhibiting superoxide dismutase (SOD), catalase, glutathione peroxidase, glutathione *S*-transferase [141,142,143], and glutathione reductase [144] in kidney tissues. Figure 4 summarizes the participation of oxidative stress in the tubular pathophysiological scenario. The results of the present study are more congruent with oxidative stress being mainly upstream of MPT and MOMP, because, after these mitochondrial events have occurred, the cell is irreversibly committed to dying [120].

Our study closely relates oxidative stress to the reduction in glomerular filtration (using pUrea as a proxy). GFR reduction is a pivotal alteration in cisplatin nephrotoxicity, derived mostly from tubular cytotoxicity (as shown in Figure 1) [2], and an internationally recognized hallmark of AKI, regardless of etiology [145]. However, tubular damage and GFR decline are not directly proportional. In fact, an undetermined degree of tubular damage may occur without affecting GFR [146,147], as undamaged nephrons may, to a certain extent, sustain (total) GFR by increasing their single-nephron GFR (SNGFR) [148]. This implies that additional injury mechanisms (unrelated to oxidative stress) might remain under maximal antioxidant circumstances. Potential oxidative stress-independent mechanisms are known (see Figure 1 and Figure 4, and [2,16,21]), but their weight in cisplatin toxicity is unknown. They would pose potential targets for pharmacological intervention in combination with antioxidants to optimize cisplatin nephrotoxicity prophylaxis. As previously reported [42], nephroprotectants whose effect lies above the model line are products showing greater protective effect than expected from their antioxidant effect. This suggests that additional protection mechanisms are involved, which makes them especially interesting candidates for clinical application. The most effective candidates include nanoceria, recombinant human erythropoietin, maltol, and the butanolic extract of *Centaurea choulettiana* Pomel (Table 2). On the contrary, those compounds lying below the model line are less effective than expected, implying that they also activate counteracting mechanisms, and are thus less interesting.

Along with its antioxidant effect, nanoceria (cerium oxide nanoparticles) also shows anti-inflammatory [101] and antiapoptotic properties [149]. Its anti-inflammatory effect has been shown to derive from the inhibition of inducible nitric oxide synthase (iNOS) expression [150] and of the NF-κB signaling pathway [151]. With regard to erythropoietin, multiple additional mechanisms have been invoked, including (i) the promotion of tubular cell regeneration, (ii) the reduction in vascular endothelial growth factor (VEGF), hemeoxygenase-1 (HO-1) and iNOS expression [89], (iii) the inactivation of macrophages via NF-κB [152], (iv) the inhibition of TGF-β1 expression [153], and (v) the reduction in polymorphonuclear cell infiltration [154]. Anti-inflammatory and antiapoptotic properties with involvement of the AMPK/PI3K/Akt pathway have also been attributed to maltol, an ingredient in the food industry [88]. Finally, traditional medicine has attributed anti-inflammatory properties to *Centaurea choulettiana* [155]. However, oxidative stress is known to be involved in the development and perpetuation of inflammation [156,157], and in the activation of apoptosis [158]. Accordingly, their anti-inflammatory and antiapoptotic properties might be the consequence of their antioxidant capacity, and would thus not explain their additional properties, which need to be further explored. Because drug discovery from plant extracts is a complicated and long process, nanoceria, erythropoietin, and maltol hoard readier potential to become clinical applications, and should thus be further explored.

## 5. Conclusions

Our results have revealed that oxidative stress is not a contributing, but a central mediator of cisplatin nephrotoxicity in preclinical models. In agreement, a recent meta-analysis identified antioxidants as the most effective protectants of cisplatin nephrotoxicity in clinical studies [159]. Interestingly, several antioxidants have shown, in animal models, nephroprotective properties without interfering with the antitumor effect of cisplatin [160,161,162,163,164], a critical issue for clinical application. This might be attributed to cisplatin genotoxicity mostly impacting on proliferating cells, such as tumor cells. In perspective, this study provides a rationale for further clinical development of preventive strategies based on single or combined therapies containing antioxidants.

## Figures and Tables

**Table 1:** Descriptive data of the studies that met the inclusion criteria. CP, cisplatin; i.p., intraperitoneal; i.v., intravenous; NPT, nephroprotectant; p.o., per os (i.e., oral administration).

Reference	Animal Species	Study Length	Cisplatin		NPT Daily Dose, Administration Route, Dose	CP + NPT (*n*)
			Dose and Route	*n*		
Abdel-Aziz et al., 2018 [50]	Rat	15 days	5 mg/kg, i.p.	8	Diacerein 50 mg/kg, p.o. 14 doses 100 mg/kg, p.o. 14 doses	8 8
Abdel Moneim et al., 2014 [51]	Rat	10 days	5 mg/kg, i.p.	7	*Azadirachta indica* leaf methanolic extract 500 mg/kg, p.o. 5 doses before CP 500 mg/kg, p.o. 5 doses after CP	7 7
Abdel-Wahab et al., 2017 [52]	Rat	4 weeks	6 mg/kg, i.p.	6	N-acetylcisteine 50 mg/kg, i.p. 12 doses Taurine 50 mg/kg, i.p. 12 doses N-acetylcisteine + Taurine 50 mg/kg, i.p. + 50 mg/kg, i.p. 12 doses	6 6 6
Alibakhshi et al., 2018 [53]	Rat	7 days	7.5 mg/kg, i.p.	5	Zingerone 50 mg/kg, p.o. 7 doses	5
Al-Husseiny et al. 2016 [54]	Rat	30 days	5 mg/kg, i.p.	20	Human amniotic fluid stem cells (5 × 10^6^)	20
Amirshahrokhi and Khalili, 2015 [55]	Mouse	4 days	15 mg/kg, i.p.	8	Thalidomide 100 mg/kg, p.o. 4 doses.	8
An et al., 2011 [56]	Mouse	5 days	5 mg/kg, i.p. once daily for 5 days	9	Pravastatin 80 mg/kg, p.o. 5 doses	9
Badawy et al., 2019 [57]	Rat	12 days	7 mg/kg, i.p.	10	Wogonin 40 mg/kg, i.p. 12 days	10
Bami et al., 2017 [58]	Rat	5 days	10 mg/kg, i.p.	6	Ferulic acid 50 mg/kg, p.o. 5 doses	6
Bayomi et al., 2013 [59]	Rat	7 days	10 mg/kg, i.p.	10	SB-4315421 mg/kg, i.p. 3 doses	10
Bazmandegan et al., 2019 [60]	Mouse	4 days	20 mg/kg, i.p	7	Sumatriptan 0.3 mg/kg, i.p. 3 doses	7
Chen et al., 2019 [61]	Mouse	3 days	20 mg/kg, i.p	5	Hesperetin 50 mg/kg, i.p. 3 doses	5
Chirino et al., 2008 [62]	Rat	10 days	7.5 mg/kg, i.p.	10	Apocynin 2 g/L in drinking water, p.o. for 10 days	10
Darwish et al., 2017 [63]	Rat	14 days	6 mg/kg, i.p.	6	Vitamin E 75 mg/kg, i.p. 14 doses	6
Dehnamaki et al., 2019 [64]	Mouse	5 days	20 mg/kg, i.p	7	Troxerutin 150 mg/kg	7
Divya et al., 2016 [65]	Rat	6 days	16 mg/kg, i.p.	6	Silymarin 100 mg/kg, p.o. 5 doses *Apodytes dimidiata* leaf methanolic extract 250 mg/kg, p.o. 5 doses before CP 250 mg/kg, p.o. 5 doses after CP	6 6 6
Elhusseini et al., 2016 [66]	Rat	30 days	10 mg/kg, i.p.	20	Human adipose-derived mesenchymal stem cells (5 × 10^6^), i.v.	20
El-Naga, 2014 [67]	Rat	2 weeks	7 mg/kg, i.p.	10	Cardamonin 10 mg/kg, p.o. 14 doses 30 mg/kg, p.o. 14 doses	10 10
El-Naga and Mahran, 2016 [7]	Rat	2 weeks	7 mg/kg, i.p.	10	Indole-3-carbinol 20 mg/kg, p.o. 14 doses	10
Elsherbiny et al., 2016 [68]	Rat	10 days	10 mg/kg, i.p.	10	Arjunolic acid 100 mg/kg, p.o. 3 doses 250 mg/kg, p.o. 3 doses	10 10
Fatima et al., 2016 [69]	Rat	6 days	7 mg/kg, i.p.	8	Epigallocatechin gallate + coenzyme Q10 15 mg/kg, i.p. + 5 mg/kg, i.p. 6 doses	8
Fernández-Rojas et al., 2014 [70]	Mouse	4 days	18 mg/kg, i.p.	4	C-phycocyanin 10 mg/kg, i.p. 1 dose 30 mg/kg, i.p. 1 dose	4 4
Hassan et al., 2014 [71]	Rat	6 weeks	7.5 mg/kg, i.p.	8	Grape seed proanthocyanidin extract 100 mg/kg, p.o. 42 doses	8
Helmy et al., 2014 [72]	Rat	4 days	6 mg/kg, i.p.	7–8	BQ-123 1 mg/kg, i.p. 2 doses	7–8
Hosseini et al., 2018 [73]	Rat	3 days	8 mg/kg, i.p.	6	*Rheum turkestanicum* root extract 100 mg/kg, i.p. 1 dose 200 mg/kg, i.p. 1 dose	6 6
Y.C. Huang et al., 2017 [74]	Mouse	3 days	20 mg/kg, i.p.	5	Galangin 75 mg/kg, p.o. 3 doses	5
H. Huang et al., 2017 [75]	Rat	12 days	8 mg/kg, i.p.	6	*Schisandra chinensis* bee pollen extract 400 mg/kg, p.o. 12 doses starting 7 days prior to CP 800 mg/kg, p.o. 12 doses starting 7 days prior to CP 1200 mg/kg, p.o. 12 doses starting 7 days prior to CP after	6 6 6
Huang et al., 2019 [76]	Mouse	4 days	22 mg/kg, i.p.	8	N-Acetylcysteine 50 mg/kg, i.p. 3 doses	8
Kandemir et al., 2019 [77]	Rat	8 days	7 mg/kg, i.p.	8	Zingerone 25 mg/kg, p.o. 7 doses 50 mg/kg, p.o. 7 doses	8 8
Kang et al., 2016 [78]	Mouse	7 days	20 mg/kg, i.p.	12	Sappanone A 10 mg/kg, i.p. 3 doses 20 mg/kg, i.p. 3 doses 40 mg/kg, i.p. 3 doses	12 12 12
Kenza et al., 2017 [79]	Mouse	11 days	8 mg/kg, i.p.	6	Vitamin E 100 mg/kg, p.o. 10 doses *Centaurea choulettiana* Pomel leaf butanolic extract 150 mg/kg, p.o. 10 doses	6 6
Khairnar et al., 2020 [80]	Rat	6 days	5 mg/kg, i.p.	6	Disulfiram 50 mM/kg, p.o. 5 doses Disulfiram + CuCl_2_ 50 mM/kg + 50 mM/kg, p.o. 5 doses Disulfiram cooper chelate (Cu-DEDC) 50 mM/kg, p.o. 5 doses Amifostine 100 mg/kg i.v. 1 dose	6 6 6 6
Kim et al., 2018 [81]	Mouse	6 days	15 mg/kg, i.p.	8	Ac-YVAD-cmk 10 mg/kg, i.p. 3 doses	8
F. Li et al., 2018 [82]	Mouse	4 days	20 mg/kg, i.p.	12	Xanthohumol 12.5 mg/kg, i.p. 3 doses 25 mg/kg, i.p. 3 doses 50 mg/kg, i.p. 3 doses	12 12 12
Y.Z. Li et al., 2018 [83]	Mouse	10 days	20 mg/kg, i.p.	8	*Schisandra chinensis* extract 300 mg/kg, p.o. 10 doses 600 mg/kg, p.o. 10 doses	8 8
Li et al., 2019 [84]	Mouse	11 days	20 mg/kg, i.p.	10	Arginyl-fructosyl-glucose 40 mg/kg, p.o. 10 doses starting 3 days prior to CP 80 mg/kg, p.o. 10 doses starting 3 days prior to CP	10 10
Ma et al., 2015 [85]	Mouse	7 days	15 mg/kg, i.p.	10	Icariin 30 mg/kg, p.o. 6 doses 60 mg/kg, p.o. 6 doses	10 10
Ma et al., 2017 [86]	Rat	5 days	7 mg/kg, i.p.	7	Puerarin 3 days before CP and 5 days after CP 30 mg/kg i.v. 50 mg/kg i.v.	7 7
Malik et al., 2015 [87]	Rat	10 days	8 mg/kg, i.p.	6	Nobiletin 5 mg/kg, i.p. 10 doses	6
Mi et al., 2018 [88]	Mouse	10	25 mg/kg i.p.	8	Maltol 100 mg/kg p.o. 10 doses starting 7 days prior to CP	8
Mohamed et al., 2013 [89]	Rat	2 weeks	9 mg/kg, i.p. divided in two doses once a week for two weeks.	20	Recombinant human erythropoietin 100 IU/kg, i.p. 14 doses	20
Morsy and Heeba, 2016 [90]	Rat	7 days	6 mg/kg, i.p.	6–8	Nebivolol 10 mg/kg, p.o. 7 doses	6–8
Mundhe et al., 2015 [91]	Rat	5 days	7.5 mg/kg, i.p.	6	Nordihydroguaiaretic acid 10 mg/kg, i.p. 5 doses	6
Mundhe et al., 2019 [92]	Rat	10 days	7.5 mg/kg i.p.	8	Nordihydroguaiarectic acid 10 mg/kg i.p. 5 doses before CP 10 mg/kg i.p. 5 doses after CP	8 8
Nazari Soltan Ahmad et al., 2018 [93]	Rat	10 days	8 mg/kg i.p.	6	Tangeretin 2.5 mg/kg i.p. 7 doses before CP and 3 after 5 mg/kg i.p. 7 doses before CP and 3 after	6 6
Nazari Soltan Ahmad et al., 2018 [94]	Rat	4 days	20 mg/kg i.p.	5	Dunnione 10 mg/kg p.o. 4 doses starting 12 h prior to CP 20 mg/kg p.o. 4 doses starting 12 h prior to CP	5 5
Neamatallah et al., 2018 [95]	Rat	11 days	7.5 mg/kg, i.p.	6	Talh honey 2.5 g/kg, p.o. 10 doses	6
Purena et al., 2018 [96]	Rat	14 days	12 mg/kg, i.p.	5	*Emblica officinalis* leaf ethanolic extract 100 mg/kg, p.o. 14 doses 200 mg/kg, p.o. 14 doses	5 5
Qi et al., 2018 [97]	Mouse	10 days	20 mg/kg i.p.	8	Pseudoginsengenin DQ 30 mg/kg p.o. 10 doses starting 7 days prior to CP 60 mg/kg p.o. 10 doses starting 7 days prior to CP	8 8
Radwan et al., 2017 [98]	Rat	10 days	7.5 mg/kg, i.p.	6	Rutin 200 mg/kg p.o. Low dose gamma radiation (LDR) Rutin 200 mg/kg p.o. + LDR	6 6 6
Rana et al., 2016 [99]	Rat	10 days	6 mg/kg, i.p.	6	*Bauhinia purpurea* bark ethanolic extract 400 mg/kg, p.o. 9 doses *Bauhinia purpurea* unripe pod extract 400 mg/kg, p.o. 9 doses	6 6
Sahin et al., 2014 [100]	Rat	12 days	7 mg/kg, i.p.	7	Curcumin difluorinated 50 mg, p.o. 12 doses Curcumin 50 mg, p.o. 12 doses	7 7
Saifi et al., 2019 [101]	Mouse	14 days 28 days	10 mg/kg i.p. 5 mg/kg i.p. CP every week for 3 consecutive weeks	6 6	Nanoceria 2 mg/kg i.p. 14 doses starting 5 days prior to CP Nanoceria 0.2 mg/kg i.p. 28 doses starting 5 days prior to 1st dose of CP 2 mg/kg i.p. 28 doses after 1st dose of CP	6 6 6
Sen et al., 2018 [102]	Rat	25 days	5 mg/kg, i.p. every five days (four injections) for 25 days	6	*Dillenia indica* fruit methanolic extract 300 mg/kg, p.o. 25 doses *Dillenia indica* fruit ethanolic extract 300 mg/kg, p.o. 25 doses	6 6
Sener et al., 2012 [103]	Rat	14 days	10 mg/kg, i.p. 14 days	7	Mirtazapine 15 mg/kg, p.o. 14 doses 30 mg/kg, p.o. 14 doses	7 7
Sharma and Goyal, 2012 [104]	Mouse	7 days	16 mg/kg, i.p.	6	*Heliotropium eichwaldii* root methanolic extract 400 mg/kg, p.o. 7 doses	6
Sherif, 2015 [105]	Rat	10 days	7 mg/kg, i.p.	10	Arjunolic acid 20 mg/kg, p.o. 10 doses	10
Shi et al., 2019 [106]	Mouse	7 days	20 mg/kg i.p.	8	Docosahexaenoic acid (DHA) 12.5 mg per day p.o. for 4 days prior to CP Docosahexaenoic acid-phosphatidylcholine (DHA-PC) 12.5 mg per day p.o. for 4 days prior to CP	8 8
Topcu-Tarladacalisir et al., 2016 [107]	Rat	10 days	7.5 mg/kg, i.p.	6	Curcumin 200 mg/kg, p.o. 4 doses	6
Z. Wang et al., 2018 [108]	Mouse	10 days	25 mg/kg i.p.	8	Ginsenoside-Re (G-Re) 25 mg/kg p.o. 10 doses starting 7 days prior to CP	8
Yang et al., 2017 [109]	Mouse	10 days	25 mg/kg, i.p.	8	Sika deer antler protein powder 10 mg/kg, p.o. 10 doses 20 mg/kg, p.o. 10 doses	8 8
Yu et al., 2018 [110]	Mouse	96 h	20 mg/kg i.p.	8	Celastrol 1 mg/kg i.p. 24 h before CP 2 mg/kg i.p. 24 h before CP	8 8
L. Zhang et al., 2018 [111]	Mouse	7 days	20 mg/kg i.p.	3	Daphnetin 2.5 mg/kg i.p. 3 doses before CP 5 mg/kg i.p. 3 doses before CP 10 mg/kg i.p. 3 doses before CP Pyrrolidine dithiocarbamate (PDTC)	3 3 3 3

**Table 2:** Products providing higher nephroprotection than expected from their antioxidant effect, according to their relative position with respect to the RANSAC linear regression model.

ID	Product	Relative Error (%)
a	Nanoceria 0.2 mg/kg (before CP) (28 doses)	>100%
b	Nanoceria 2 mg/kg (after CP) (28 doses)	>75%
c	RH Erythropoietin 100 IU/kg	>75%
d	Maltol 100 mg/kg	>50%
e	*Centaurea choulettiana* Pomel leaf butanolic extract 150 mg/kg	>50%
f	Wogonin 40 mg/kg	>25%
g	Rutin 200 mg/kg	>25%
h	Celastrol 1 mg/kg	>25%
i	SB-431542 1 mg/kg	>25%
j	Arjunolic acid 100 mg/kg	>25%
k	Troxerutin 150 mg/kg	>25%
l	Sika deer antler protein powder 10 mg/kg	>25%
m	Puerarin 50 mg/kg	>25%
*n*	N-acetylcisteine 50 mg/kg	>25%
o	Mesenchymal stem cells 5 × 10^6^ cells	>25%
p	Human amniotic fluid stem cells 5 × 10^6^ cells	>25%
q	Vitamin E 100 mg/kg	>25%
r	Sumatriptan 0.3 mg/kg	>25%
s	Celastrol 2 mg/kg	>25%
t	Taurine 50 mg/kg	>25%
u	Daphnetin 2.5 mg/kg	>25%
v	Sappanone A 10 mg/kg	>25%

## Data Availability

The data presented in this study are available in the article.
